# Relationship of Genetic Polymorphisms of the Chemokine, CCL5, and Its Receptor, CCR5, with Coronary Artery Disease in Taiwan

**DOI:** 10.1155/2015/851683

**Published:** 2015-11-24

**Authors:** Ke-Hsin Ting, Kwo-Chang Ueng, Whei-Ling Chiang, Ying-Erh Chou, Shun-Fa Yang, Po-Hui Wang

**Affiliations:** ^1^Institute of Medicine, Chung Shan Medical University, Taichung 402, Taiwan; ^2^Division of Cardiology, Department of Internal Medicine, Yuan-Sheng Hospital and Changhua Christian Hospital, Yuanlin Branch, Yuanlin 510, Taiwan; ^3^Department of Internal Medicine, Chung Shan Medical University Hospital, Taichung 402, Taiwan; ^4^School of Medicine, Chung Shan Medical University, Taichung 402, Taiwan; ^5^School of Medical Laboratory and Biotechnology, Chung Shan Medical University, Taichung 402, Taiwan; ^6^Department of Medical Research, Chung Shan Medical University Hospital, Taichung 402, Taiwan; ^7^Department of Forensic Medicine, Chung Shan Medical University, Taichung 402, Taiwan; ^8^Department of Obstetrics and Gynecology, Chung Shan Medical University Hospital, Taichung 402, Taiwan

## Abstract

The chemokine receptor CCR5 polymorphism, which confers resistance to HIV infection, has been associated with reduced risk of cardiovascular disease. However, the association of the chemokine, CCL5, and its receptor, CCR5, polymorphism and coronary artery disease (CAD) in the Taiwanese has not been studied. In this study, 483 subjects who received elective coronary angiography were recruited from Chung Shan Medical University Hospital. CCL5-403 and CCR5-59029 were determined by polymerase chain reaction-restriction fragment length polymorphism. We found that CCL5-403 with TT genotype frequencies was significantly associated with the risk of CAD group (odds ratio = 3.063 and *p* = 0.012). Moreover, the frequencies of CCR5-59029 with GG or GA genotype were higher than AA genotype in acute coronary syndrome individuals (odds ratio = 1.853, CI = 1.176–2.921, *p* = 0.008). In conclusion, we found that CCL5-403 polymorphism may increase genetic susceptibility of CAD. CCL5-403 or CCR5-59029 single nucleotide polymorphism may include genotype score and it may predict cardiovascular event.

## 1. Introduction

The importance of coronary artery disease (CAD) in contemporary society is attested to by the almost epidemic number of persons [1]. The World Health Organization (WHO) has estimated that by 2020 the global number of deaths from CAD will have risen from 7.2 million in 2002 to 11.2 million [2]. In Taiwan, ischemic heart disease had become the second etiology of all-cause mortality in the recent 10 years. Based on database from the SCORE system or Framingham Heart study, the cardiovascular risk of developing symptomatic CAD after age of 40 is 49 percentage for man and 32 percentage for women [3, 4]. The conventional risk factors are age, smoking, hypertension, diabetes, dyslipidemia, or metabolic syndrome. Despite the importance of hyperlipidemia, half of all myocardial infarctions occur in the patients without overt hyperlipidemia. Although the use of global prediction models like Framingham risk sore greatly improves the detection of heart disease risk [4], as many as 20 percentage of all events occur in the absence of any of the major classic cardiovascular risk sores. Therefore, a serial novel risk factor has developed in the recent decades, including hsCRP, IL-6, IL-18, homocysteine, or chemokines [5].

Chemokines (chemotactic cytokines) are small heparin-binding proteins that direct the movement of circulating leukocytes to sites of inflammation or atherosclerosis [6]. The chemokines comprise a family of small (8~14 kDa) highly basic proteins with similar tertiary structure. They are divided into subfamilies on the basis of the number and sequential relationship of their conserved cysteine residues (CXC, CC, CX3C, and XC subfamilies). Chemokines affect cells by activating surface receptors that are seven-transmembrane-domain G-protein-coupled receptors. Leukocyte responses to particular chemokines are determined by their complement of chemokine receptors [7]. Additionally, genome-wide association studies (GWAS) have identified a large number of robust associations between special chromosomal loci and complex human disease [8]. Determination of single nucleotide polymorphisms (SNPs) and haplotype blocks has led to increasingly effective approaches to the identification of genetic variation associated with various multifactorial diseases inclusive of chemokines study.

CCR5 was famous as a coreceptor for macrophage-tropic HIV-1 [9]. A 32-base-pair deletion polymorphism in CCR5, known as CCR5 Δ32, was recognized and found to lead to near complete resistance to HIV-1 infection in the homozygous state and slower progression to AIDS in the heterozygous state [10, 11]. CCR5 Δ32 allele has been linked with reduced susceptibility to coronary artery disease [12]. CCR5 may be more important in the later stage plaque development [13]. CCR5 immunoreactivity is also detected in human unstable atherosclerotic plaque with upregulation of CCR5 mRNA expression [14]. Moreover, CCR5 Δ32 polymorphism has been found to associate with the higher high density lipoprotein (HDL) and lower triglycerides (TG) level [15]. Besides, the ligands of CCR5, including CCL5 (regulated upon activation, normal T-cell expressed and secreted, RANTES), have the important role in the initiation and progression of atherosclerosis. CCL5 acting at CCR5 is considered to be crucial to monocyte recruitment during development of atherosclerosis. Knock-down of CCR5 expression in vascular smooth muscle was found to reduce neointimal thickening and macrophage infiltration in mouse model [16]. Use of Met-RANTES, which prevents CCL5 interaction with glycosaminoglycans and so inhibits its function, was found to lessen progression of atherosclerotic plaque [17].

In the recent decades, many serial studies about single nucleotide polymorphism of CCR5 and its ligand were published. However, the results of the different studies were diversity. CCR5 Δ32 had been identified as the reduced early onset coronary artery disease in women [18]. In the study of Szalai et al., CCR5 Δ32 genotype was protective against CAD compared with no CCR5 Δ32 homozygotes in CAD patients [19]. However, there was no effect of CCR5 Δ32 polymorphism on coronary artery disease or acute coronary syndrome in other studies. In the Nurses' Health Study, the authors found no association between CCR5 Δ32 polymorphism or five other CCR5 polymorphisms and the risk of CAD [18]. In the study of González et al., they found a strong inverse association for CCR5 Δ32 polymorphism and early age of CAD onset [20]. These results may reflect differences in the populations, including variations in the frequency of the CCR5 Δ32 allele. For example, the prevalence of CCR55 Δ32 allele in Europe is approximately 10%, and it is low or absent in most of the Asian and African populations [21].

CCL5 (RANTES) is one of the CCR5 ligands that have been linked to atherosclerosis. Simeoni et al. reported that RANTES A-403 was associated with CAD independently of conventional factors and hsCRP or fibrinogen as inflammatory biomarkers [22]. The association was enhanced in smokers and acute coronary syndrome. Moreover, in the other report of Böger et al., CCL5-403 polymorphism was associated with all-cause mortality, mainly due to cardiac event, in patients with type 2 diabetes and end stage renal disease [23]. However, Szalai et al. found no association between the CCL5 polymorphism -28G and -403A and CAD [19].

In the Asian study, Nakajima et al. found that the CCL5 promoter-28G genotype and CCR5 promoter 59029A genotype may be independent risk factors for diabetic nephropathy and may have an additive effect on nephropathy in the Japanese populations [24]. However, the influence of CCL5 A-403 and CCR5 59029A polymorphism on coronary artery disease has not been identified in Taiwan's population. The frequency of the two polymorphisms was more popular than CCR5 Δ32 polymorphism in Asian. Therefore, our study was focused on the association between coronary artery disease with CCL5 A-403 and CCR5 59029A polymorphism.

## 2. Materials and Methods

### 2.1. Study Population

There were 483 Taiwanese adult patients with angina pectoris included in this study during the period from April 2007 through March 2009 in the Chung Shan Medical University Hospital. All of them received coronary angiography after the serial cardiovascular noninvasive examinations. The exclusive criteria were recent stroke, out-hospital cardiac arrest, and incomplete data. They were divided to CAD group (lumen stenosis > 50%; 322 patients) or non-CAD group (lumen stenosis < 50%; 161 patients) according to the result of coronary angiography. There were 348 male (108 non-CAD and 240 CAD) and 135 female (53 non-CAD and 82 CAD) patients in our study. All the patients were given an informed consent and were well told of the study protocol. Blood samples were collected via venous puncture and were analyzed by the central research laboratory. The study was approved by the Institutional Review Board of Chung Shan Medical University Hospital (CSMUH number CS07095), and informed consent was obtained from each participant.

### 2.2. Angiographic Classification

Coronary angiography was performed via transradial or transfemoral approach. The lumen stenosis of coronary artery was measured by quantitative coronary angiography (QCA). Angiographic criteria defining CAD cases or controls were as follows: (1) cases group: lumen stenosis > 50% lumen narrowing on one or more major epicardial coronary arteries, (2) control group: lumen stenosis < 50% lumen narrowing on one or more major epicardial coronary artery segments.

### 2.3. Polymerase Chain Reaction-Restriction Fragment Length Polymorphism (PCR-RFLP)

**Figure 1:**
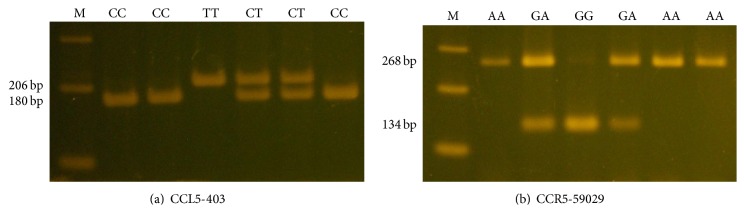
Polymerase chain reaction-restriction fragment length polymorphisms of CCL5-403 and CCR5-59029 genes. (a) PCR products of CCL5-403 gene polymorphisms were subjected to enzymatic digestion by incubation with Rsa I, for 4 hours at 37°C, and then submitted to electrophoresis in 3% agarose gels. The wild homozygous alleles (C/C) yielded a 206-bp product, and the heterozygous alleles (C/T) yielded 206-, 180-, and 26-bp products, while the mutant homozygous alleles (T/T) yielded 180- and 26-bp products. (b) PCR products of the CCR5-59029 gene polymorphism were subjected to enzymatic digestion by incubation with Bsp 1286I. The wild homozygous alleles (G/G) yielded 134-bp products, and the heterozygous alleles (G/A) yielded 268- and 134-bp products, while the mutant homozygous alleles (A/A) yielded a 268-bp product.

The information of reference site in CCL5-403 and CCR5-59029 was provided to our study from the website of National Center Biotechnology Information (NCBI). Genotyping for CCL5-403 and its receptor CCR5-59029 was determined by PCR-restriction fragment length polymorphism assay (PCR-RFLP). Sequences of primers used for analysis of CCL5-403 rs2107538 C/T genotype were 5′-CACAAGAGGACTCATTCCAACTCA-3′ (forward primer) and 5′-GTTCCTGCTTATTCATTACAGATCGTA-3′ (reverse primer) to yield a product of 206 bps. The CCR5-59029 rs1799987 G/A polymorphism was amplified with the following primer: 5′-CCCGTGAGCCCATAGTTAAAACTC-3′ (forward primer) and 5′-TCACAGGGCTTTTCAA CAGTAAGG-3′ (reverse primer) (268 bp). We used the endonucleases Rsa I and Bsp 1286I for restricted enzyme. Polymerase chain reaction was performed in a 10 *μ*L volume containing 100 ng DNA template, 1.0 *μ*L of 10x PCR buffer (Invitrogen, Carlsbad, CA), 0.25 U of Taq DNA polymerase (Invitrogen), 0.2 mM dNTPs (Promega, Madison, WI), and 200 nM of each primer (MDBio Inc., Taipei, Taiwan). The PCR cycling conditions were performed: 94°C for 5 min, followed by 35 cycles of 1 min at 94°C, 1 min at 57°C, and 2 min at 72°C, with a final step at 72°C for 20 min to allow a complete extension of all PCR fragments. For CCL5-403, the wild homozygous alleles (C/C) yielded a 206-bp product, and the heterozygous alleles (C/T) yielded 206-, 180-, and 26-bp products, while the mutant homozygous alleles (T/T) yielded 180- and 26-bp products. For CCR5-59029, the wild homozygous alleles (G/G) yielded 134-bp products, and the heterozygous alleles (G/A) yielded 268- and 134-bp products, while the mutant homozygous alleles (A/A) yielded a 268-bp product (Figure 1). Moreover, the genotypes determined by PCR-RFLP were confirmed by DNA electrophoresed sequencing analysis.

### 2.4. Statistical Analysis

The average age is presented as the mean ± SD. A Mann-Whitney *U* test and Fisher's exact test were used to compare the differences of age as well as demographic characteristics distributions between control group and cases group with CAD, since the small sample size was present in some categorical variables. The odds ratios (ORs) with their 95% confidence intervals (CIs) of the association between genotype frequencies and CAD susceptibility as well as clinical characteristics were estimated by multiple logistic regression models. A *p* value < 0.05 was considered significant. The data were analyzed on SAS statistical software (Version 9.1, 2005; SAS Institute Inc., Cary, NC). We assessed Hardy-Weinberg equilibrium by using a goodness-of-fit *χ* ^2^ test for biallelic markers.

## 3. Results

The clinical characteristics of this study were summarized in Table 1. According to the results of angiography, 483 individuals were divided into cases group with coronary artery disease (*n* = 322) and control group (*n* = 161). In the two groups, there were no significant differences in gender, age, height, weight, smoking, family history, hyperlipidemia, and diabetes. However, significant differences were observed between case group and control group for characteristics like hypertension, atrial fibrillation, high CAD risk factors, acute coronary syndrome, aspirin used, heart failure, and recent angina (*p* < 0.05).

The genotype frequencies and distribution in cases and control groups were shown in Table 2. Both groups were in Hardy-Weinberg equilibriums. TT homozygote frequency of CCL5-403 was significantly different between cases and controls (*p* = 0.012). TT homozygote of CCL5-403 was 3.063-fold significant increasing risk in CAD group compared to CC or CT individuals (95% CI = 1.231–7.624, *p* = 0.012). T allele frequency of CCL5-403 was also significantly different between cases and controls (OR: 1.377; 95% CI = 1.019–1.859, *p* = 0.037). However, GA or AA genotype frequency of CCR5-59029 did not show any statistically significant difference (odds ratio = 1.026, CI = 0.699–1.506, *p* = 0.896). An allele frequency of CCR5-59029 was not found significantly different among the cases group and control group (odds ratio = 1, CI = 0.757–1.321).

We further analyzed the association between CCL5-403, CCR5-59029 genotype, and the demographics features in Table 3. There was no correlation between CCL5-403, CCR5-59029 genotype, and conventional factors like age, BMI, SBP, or DBP.

We also further analyzed the association between CCL5-403, CCR5-59029 genotype, and the clinical characteristics in Tables 4 and 5. There was no correlation between CCL5-403 genotype and clinical characteristics, such as AF positive, hypertension, diabetes, dyslipidemia, and elevated cardiac enzyme. However, we observed the frequencies of GA or AA genotype higher than GG genotype in acute coronary syndrome individuals (odds ratio = 1.853, CI = 1.176–2.921, *p* = 0.008).

## 4. Discussion

The chemokine receptor CCR5 was well known as the coreceptor for HIV infection of macrophages. CCR5 deletion had reported the resistance to HIV infection and was found to reduce cardiovascular risk in animal models [25]. CCL5 acting at CCR5 was considered to be crucial to monocyte recruitment during the early atherosclerosis [26]. CCL5 was released from platelet *α*-granules together with PF-4/CXCL4 and deposited on the inflamed endothelium [27]. CCL5 antagonist inhibited atherosclerosis progression in mouse models [28]. Gene polymorphism was the hint to find the biological mechanism of chemokines and their cellular receptors may influence monocytes trafficking in the atherosclerosis process.

In our case control study, TT homozygote of CCL5-403 was 3.063-fold significant increasing risk in CAD group compared to CC or CT individuals (95% CI = 1.231–7.624, *p* = 0.012). T allele odds ratio of CCL5-403 was 1.377-fold significant increasing risk in CAD group compared to C allele (95% CI = 1.019–1.859, *p* = 0.037). The results were compatible with the findings of Simeoni et al. [22]. They found that CCL5 A-403 was associated with CAD independently of the conventional risk factors. CCL5 A-403 may increase genetic susceptibility to CAD. We found that the results were the same in Asian and Caucasian without limited ethnic heterogeneity. However, the protective effect of CCR5 deletion (Δ32 allele) against early myocardial infarction was diversity in Spanish report [20] and Indian report [29]. Most importantly, the prevalence of CCR5 deletion (Δ32 allele) in Europe was approximately 10%, but it was low or absent in most of the Asian African populations. Therefore, CCL5-403 polymorphism was the important SNPs in Asian. Additionally, we observed the frequencies of GG or GA genotype of CCR5-59029 higher than AA genotype of CCR5-59029 in acute coronary syndrome individuals (odds ratio = 1.853, CI = 1.176–2.921, *p* = 0.008). The association between CCR5-59029 and acute coronary syndrome was not shown in other reports. GA or AA genotype frequency of CCR5-59029 did not show any statistically significant difference. Thus, we assumed CCR5-59029 polymorphism was associated with acute coronary syndrome but not chronic stable coronary artery disease. Additionally, knock-down CCL5 expression in vascular muscle was found to reduce neointimal thickening and macrophage infiltration in mouse model [16]. In summary, the role of CCL5 was more important than CCR5 for atherosclerosis.

There were many chemokines and their receptors play a role in the development of atherosclerosis. Specially, evidence was established as the important roles and key plays for CCL2/CCR2, CX3CL1/CX3CR1, and CCL5/CCR5 [25]. The polymorphism in MCP-1 G-2518 had been associated with increased transcription of MCP-1(CCL2) gene. Szalai et al. found that the frequency of MCP-1 G-2518 homozygote variant was significantly higher in CAD group [19]. The rare Val64Ile polymorphism in CCR2 gene coding for MCP-1 receptor was associated with reduced coronary calcification in the other study [30]. Fractalkine (CX3CL1) and its receptor, CX3CR1, which are expressed in human plaques, had been implicated in atherosclerosis. CX3CR1I-249 heterozygote was associated with markedly reduced risk of acute coronary syndrome [31]. In the Framingham Heart Cohort study [32], CX3CR1 M-280 polymorphism was independently associated with a low risk of cardiovascular disease. Therefore, the correlated interaction between several different chemokines and their receptors might be present in atherosclerosis. In brief, the polymorphism of CCL2/CCR2, CX3CL1/CX3CR1, and CCL5/CCR5 should be detected simultaneously for the comprehensive study.

There were numerous studies investigating whether serum CCL5 levels were associated with cardiovascular disease, although it should be noted that levels of circulating chemokine do not correspond to levels in tissue. In Kraaijeveld et al.'s study, high circulating levels of CCL5 might be a marker for unstable angina [33]. In contrast, lower serum CCL5 was found to be associated with coronary heart disease [34]. Additionally, the use of heparin in acute coronary syndrome might lead to increased CCL5 levels due to the release of heparin sulfate-bound chemokines in the vessel wall [35]. Thus, the instability of CCL5 levels became its unreliable maker of coronary atherosclerosis. Then, genetic polymorphisms in chemokine genes were better risk factors for atherosclerosis than chemokine levels.

The limitations in this study are shown below. Because we divided all patients to cases group and control group by the result of coronary angiography, allocation bias may be found in this study. For example, the percentage of atrial fibrillation in cases group is only 9.3% and the result is very lower than the 24–46% prevalence of atrial fibrillation in the previous report [36]. 24 hour Holter electrocardiogram may be considered to confirm the diagnosis. Additionally, the percentage of heart failure in CAD group is lower than control group (21.3% versus 32.9%). Because systolic heart failure is associated with coronary heart disease in contrast to diastolic heart failure, we should divide heart failure to these different types in clinical condition [37]. On the other hand, the limitations of the polymorphism studies are needed to be tested and validated. There is the potential possibility for generating false-positive or false-negative results and for biases related to the selection of study participants and genotyping errors [38]. Besides, some of chemokines and their receptor genotypes are rare, making epidemiological conclusions difficult and diverse in different races.

In conclusion, CCL5-403 polymorphism may increase genetic susceptibility of CAD. CCL5-403 with TT genotype or T allele is associated with CAD. CCR5-59029 polymorphism is associated with acute coronary syndrome but not chronic stable coronary artery disease. The SNPs of CCL5/CCR5 can be the predictive chemokine factor except for CCL2/CCR2 or CX3CL1/CX3CR1 polymorphism in the Asian population.

## Figures and Tables

**Table 1:** Demographics and clinical characteristics of patients in non-CAD (*n* = 161) and CAD groups (*n* = 322)^1,2^.

	Non-CAD (*n* = 161)	CAD (*n* = 322)	*p* value
Gender			
Male	108 (67.1%)	240 (74.5%)	0.085
Age (y)	65.79 ± 12.41	66.69 ± 11.00	0.929
Height (cm)	161.28 ± 8.92	161.85 ± 8.49	0.499
Weight (kg)	65.99 ± 13.21	66.91 ± 12.62	0.461
BMI (kg/m^2^)	25.26 ± 3.92	25.52 ± 4.50	0.525
AF positive (%)	41 (25.5%)	30 (9.3%)	<0.001
CAD risk ≥3 (%)	76 (47.2%)	190 (59.0%)	0.014
Age >65 year (%)	90 (55.9%)	174 (54.0%)	0.698
Family history (%)	34 (21.1%)	59 (18.3%)	0.463
Hypertension (%)	95 (59.0%)	242 (75.2%)	<0.001
Diabetes mellitus (%)	56 (34.8%)	139 (43.2%)	0.077
Active smoker (%)	64 (39.8%)	146 (45.3%)	0.243
Cholesterol >200 (%)	66 (41.8%)	129 (40.3%)	0.760
ASA use in the past 7 days (%)	39 (24.4%)	117 (38.2%)	0.003
Recent (<24 h) sever angina (%)	90 (55.9%)	227 (70.7%)	0.001
Cardiac markers elevation (%)	66 (41.0%)	194 (60.2%)	<0.001
Stroke	21 (13.8%)	34 (11.0%)	0.374
CHF	52 (32.9%)	67 (21.3%)	0.006
SBP (mmHg)	130.56 ± 18.64	132.86 ± 21.13	0.246
DBP (mmHg)	77.61 ± 14.31	79.28 ± 15.62	0.260

^1^Data were presented as number (percentage) with chi-square test/Fisher exact test.

^2^Mean ± SD with independent two-sample *t*-test.

**Table 2:** Odds ratio (OR) and 95% confidence interval (CI) of CAD patients associated with genotyping frequencies of CCL5-403 and CCR5-59029.

Variable	Non-CAD (*n* = 161) (%)	CAD (*n* = 322) (%)	OR (95% CI)	*p* value
CCL5-403				
CC	85 (52.8%)	148 (46.0%)	1.00	
CT	70 (43.5%)	142 (44.1%)	1.165 (0.788–1.723)	0.444
TT	6 (3.7%)	32 (9.9%)	3.063 (1.231–7.624)	0.012^*∗*^
CC	85 (52.8%)	148 (46.0%)	1.00	
CT or TT	76 (47.2%)	174 (54.0%)	1.315 (0.900–1.921)	0.157
C	240 (74.5%)	438 (68.0%)	1.00	
T	82 (25.5%)	206 (32.0%)	1.377 (1.019–1.859)	0.037^*∗*^
CCR5-59029				
GG	67 (41.6%)	132 (41.0%)	1.00	
GA	71 (44.1%)	146 (45.3%)	1.044 (0.694–1.570)	0.837
AA	23 (14.3%)	44 (13.7%)	0.971 (0.542–1.741)	0.921
GG	67 (41.6%)	132 (41.0%)	1.00	
GA or AA	94 (58.4%)	190 (59.0%)	1.026 (0.699–1.506)	0.896
G	205 (63.7%)	410 (63.7%)	1.00	
A	117 (36.3%)	134 (36.3%)	1.000 (0.757–1.321)	1.000

The odds ratio (OR) with their 95% confidence intervals were estimated by logistic regression.

^*∗*^Significance *p* value < 0.05.

**Table 3:** Association of demographics features between two alleles of CCL5-403 and CCR5-59029 for CAD group^1^.

	CCL5-403	CCL5-403	*p* value
	CC (*n* = 148)	CT + TT (*n* = 174)	
Age (y)	65.28 ± 10.80	66.03 ± 11.18	0.542
Height (cm)	162.00 ± 9.13	161.71 ± 7.90	0.759
Weight (kg)	67.50 ± 13.37	66.41 ± 11.95	0.440
BMI (kg/m^2^)	25.71 ± 4.94	25.37 ± 4.09	0.504
SBP (mmHg)	134.60 ± 22.14	131.41 ± 20.20	0.180
DBP (mmHg)	80.97 ± 16.09	77.85 ± 15.10	0.076

	CCR5-59029	CCR5-59029
	GG (*n* = 132)	GA + AA (*n* = 190)

Age (y)	66.00 ± 10.83	65.47 ± 11.13	0.673
Height (cm)	161.86 ± 8.03	161.84 ± 8.80	0.986
Weight (kg)	66.88 ± 13.50	66.93 ± 11.99	0.971
BMI (kg/m^2^)	25.45 ± 4.36	25.58 ± 4.60	0.798
SBP (mmHg)	131.72 ± 19.82	133.67 ± 22.02	0.419
DBP (mmHg)	78.67 ± 14.90	79.70 ± 16.12	0.565

^1^Mean ± SD with independent two-sample *t*-test.

**Table 4:** Genotyping frequencies and association of demographics features between two alleles of CCL5-403 for CAD group^1^.

	CCL5-403	CCL5-403	OR (95% CI)	*p* value
	CC (*n* = 148)	CT + TT (*n* = 174)		
Male	110 (74.3%)	130 (74.7%)	1.021 (0.617–1.688)	0.936
AF positive (%)	15 (10.1%)	15 (8.6%)	0.836 (0.394–1.774)	0.641
CAD risk ≥3 (%)	85 (57.4%)	105 (60.3%)	1.128 (0.722–1.761)	0.596
Age >65 year (%)	72 (48.6%)	102 (58.6%)	1.495 (0.962–2.325)	0.074
Family history (%)	29 (19.6%)	30 (17.2%)	0.855 (0.486–1.504)	0.856
Hypertension (%)	114 (77.0%)	128 (73.6%)	0.830 (0.498–1.382)	0.473
Diabetes mellitus (%)	69 (46.6%)	70 (40.2%)	0.771 (0.495–1.200)	0.248
Active smoker (%)	68 (45.9%)	78 (44.8%)	0.956 (0.616–1.485)	0.841
Cholesterol >200 (%)	58 (39.2%)	71 (41.3%)	1.091 (0.697–1.708)	0.704
ASA use in the past 7 days (%)	62 (43.7%)	55 (33.5%)	0.651 (0.409–1.035)	0.069
Recent (<24 h) sever angina (%)	103 (69.6%)	124 (71.7%)	1.106 (0.683–1.790)	0.683
Cardiac markers elevation (%)	90 (60.8%)	104 (59.8%)	0.957 (0.612–1.499)	0.849
Stroke	16 (11.4%)	18 (10.6%)	0.918 (0.449–1.874)	0.814
CHF	30 (21.1%)	37 (21.4%)	1.016 (0.590–1.747)	0.955

^1^Data were presented as number (percentage) with chi-square test/Fisher exact test.

**Table 5:** Genotyping frequencies and association of demographics features between two alleles of CCR5-59029 for CAD group^1^.

	CCR5-59029	CCR5-59029	OR (95% CI)	*p* value
	GG (*n* = 132)	GA + AA (*n* = 190)		
Male	96 (72.7%)	144 (75.8%)	1.174 (0.707–1.949)	0.535
AF positive (%)	13 (9.8%)	17 (8.9%)	0.900 (0.421–1.921)	0.784
CAD risk ≥3 (%)	80 (60.6%)	110 (57.9%)	0.894 (0.568–1.405)	0.627
Age >65 year (%)	74 (56.1%)	100 (52.6%)	0.871 (0.557–1.361)	0.544
Family history (%)	26 (19.7%)	33 (17.4%)	0.857 (0.485–1.515)	0.595
Hypertension (%)	95 (72.0%)	147 (77.4%)	1.331 (0.800–2.216)	0.270
Diabetes mellitus (%)	65 (49.2%)	74 (38.9%)	0.658 (0.420–1.030)	0.067
Active smoker (%)	54 (40.9%)	92 (48.4%)	1.356 (0.866–2.124)	0.183
Cholesterol >200 (%)	57 (43.5%)	72 (38.1%)	0.799 (0.508–1.257)	0.331
ASA use in the past 7 days (%)	53 (42.7%)	64 (35.2%)	0.727 (0.455–1.160)	0.181
Recent (<24 h) sever angina (%)	86 (65.2%)	141 (74.6%)	1.571 (0.967–2.553)	0.067
Cardiac markers elevation (%)	68 (51.5%)	126 (66.3%)	1.853 (1.176–2.921)	0.008^*∗*^
Stroke	16 (12.6%)	18 (9.8%)	0.757 (0.370–1.547)	0.444
CHF	30 (23.1%)	37 (20.0%)	0.833 (0.484–1.436)	0.511

^1^Data were presented as number (percentage) with chi-square test/Fisher exact test.

^*∗*^Significance *p* value < 0.05.

## References

[1] Kowalchuk G. J., Siu S. C., Lewis S. M. (1990). Coronary artery disease in the octogenarian: angiographic spectrum and suitability for revascularization. *The American Journal of Cardiology*.

[2] Mason J. C., Libby P. (2014). Cardiovascular disease in patients with chronic inflammation: mechanisms underlying premature cardiovascular events in rheumatologic conditions. *European Heart Journal*.

[3] Reiner Z., Catapano A. L., De Backer G. (2011). ESC/EAS Guidelines for the management of dyslipidaemias: the Task Force for the management of dyslipidaemias of the European Society of Cardiology (ESC) and the European Atherosclerosis Society (EAS). *European Heart Journal*.

[4] Wilson P. W. F., D'Agostino R. B., Levy D., Belanger A. M., Silbershatz H., Kannel W. B. (1998). Prediction of coronary heart disease using risk factor categories. *Circulation*.

[5] Weber C., Noels H. (2011). Atherosclerosis: current pathogenesis and therapeutic options. *Nature Medicine*.

[6] Frangogiannis N. G. (2008). The immune system and cardiac repair. *Pharmacological Research*.

[7] Charo R. M., Ransohoff R. M. (2006). The many roles of chemokines and chemokine receptors in inflammation. *The New England Journal of Medicine*.

[8] Hardy J., Singleton A. (2009). Genomewide association studies and human disease. *The New England Journal of Medicine*.

[9] Combadiere C., Ahuja S. K., Tiffany H. L., Murphy P. M. (1996). Cloning and functional expression of CC CKR5, a human monocyte CC chemokine receptor selective for MIP-1*α*, MIP-1*β*, and RANTES. *Journal of Leukocyte Biology*.

[10] Dean M., Carrington M., Winkler C. (1996). Genetic restriction of HIV-1 infection and progression to AIDS by a deletion allele of the *CKR5* structural gene. *Science*.

[11] Huang Y., Paxton W. A., Wolinsky S. M. (1996). The role of a mutant CCR5 allele in HIV-1 transmission and disease progression. *Nature Medicine*.

[12] Afzal A. R., Kiechl S., Daryani Y. P. (2008). Common CCR5-del32 frameshift mutation associated with serum levels of inflammatory markers and cardiovascular disease risk in the Bruneck population. *Stroke*.

[13] Quinones M. P., Martinez H. G., Jimenez F. (2007). CC chemokine receptor 5 influences late-stage atherosclerosis. *Atherosclerosis*.

[14] Papaspyridonos M., Smith A., Burnand K. G. (2006). Novel candidate genes in unstable areas of human atherosclerotic plaques. *Arteriosclerosis, Thrombosis, and Vascular Biology*.

[15] Hyde C. L., MacInnes A., Sanders F. A. (2010). Genetic association of the CCR5 region with lipid levels in at-risk cardiovascular patients. *Circulation: Cardiovascular Genetics*.

[16] Krohn R., Raffetseder U., Bot I. (2007). Y-box binding protein-1 controls CC chemokine ligand-5 (CCL5) expression in smooth muscle cells and contributes to neointima formation in atherosclerosis-prone mice. *Circulation*.

[17] Braunersreuther V., Steffens S., Arnaud C. (2008). A novel RANTES antagonist prevents progression of established atherosclerotic lesions in mice. *Arteriosclerosis, Thrombosis, and Vascular Biology*.

[18] Pai J. K., Kraft P., Cannuscio C. C. (2006). Polymorphisms in the CC-chemokine receptor-2 (CCR2) and -5 (CCR5) genes and risk of coronary heart disease among US women. *Atherosclerosis*.

[19] Szalai C., Duba J., Prohászka Z. (2001). Involvement of polymorphisms in the chemokine system in the susceptibility for coronary artery disease (CAD). Coincidence of elevated Lp(a) and MCP-1 −2518 G/G genotype in CAD patients. *Atherosclerosis*.

[20] González P., Alvarez R., Batalla A. (2001). Genetic variation at the chemokine receptors CCR5/CCR2 in myocardial infarction. *Genes and Immunity*.

[21] Lucotte G. (2002). Frequencies of 32 base pair deletion of the (Δ32) allele of the CCR5 HIV-1 co-receptor gene in Caucasians: a comparative analysis. *Infection, Genetics and Evolution*.

[22] Simeoni E., Winkelmann B. R., Hoffmann M. M. (2004). Association of RANTES G-403A gene polymorphism with increased risk of coronary arteriosclerosis. *European Heart Journal*.

[23] Böger C. A., Fischereder M., Deinzer M. (2005). RANTES gene polymorphisms predict all-cause and cardiac mortality in type 2 diabetes mellitus hemodialysis patients. *Atherosclerosis*.

[24] Nakajima K., Tanaka Y., Nomiyama T. (2003). RANTES promoter genotype is associated with diabetic nephropathy in type 2 diabetic subjects. *Diabetes Care*.

[25] Jones K. L., Maguire J. J., Davenport A. P. (2011). Chemokine receptor CCR5: from AIDS to atherosclerosis. *British Journal of Pharmacology*.

[26] Lutgens E., Faber B., Schapira K. (2005). Gene profiling in atherosclerosis reveals a key role for small inducible cytokines: validation using a novel monocyte chemoattractant protein monoclonal antibody. *Circulation*.

[27] von Hundelshausen P., Koenen R. R., Sack M. (2005). Heterophilic interactions of platelet factor 4 and RANTES promote monocyte arrest on endothelium. *Blood*.

[28] Veillard N. R., Kwak B., Pelli G. (2004). Antagonism of RANTES receptors reduces atherosclerotic plaque formation in mice. *Circulation Research*.

[29] Sharda S., Gilmour A., Harris V. (2008). Chemokine receptor 5 (CCR5) deletion polymorphism in North Indian patients with coronary artery disease. *International Journal of Cardiology*.

[30] Ortlepp J. R., Vesper K., Mevissen V. (2003). Chemokine receptor (CCR2) genotype is associated with myocardial infarction and heart failure in patients under 65 years of age. *Journal of Molecular Medicine*.

[31] Moatti D., Faure S., Fumeron F. (2001). Polymorphism in the fractalkine receptor CX3CR1 as a genetic risk factor for coronary artery disease. *Blood*.

[32] McDermott D. H., Fong A. M., Yang Q. (2003). Chemokine receptor mutant CX3CR1-M280 has impaired adhesive function and correlates with protection from cardiovascular disease in humans. *The Journal of Clinical Investigation*.

[33] Kraaijeveld A. O., de Jager S. C. A., de Jager W. J. (2007). CC chemokine ligand-5 (CCL5/RANTES) and CC chemokine ligand-18 (CCL18/PARC) are specific markers of refractory unstable angina pectoris and are transiently raised during severe ischemic symptoms. *Circulation*.

[34] Rothenbacher D., Müller-Scholze S., Herder C., Koenig W., Kolb H. (2006). Differential expression of chemokines, risk of stable coronary heart disease, and correlation with established cardiovascular risk markers. *Arteriosclerosis, Thrombosis, and Vascular Biology*.

[35] Ranjbaran H., Wang Y., Manes T. D. (2006). Heparin displaces interferon-*γ*-inducible chemokines (IP-10, I-TAC, and Mig) sequestered in the vasculature and inhibits the transendothelial migration and arterial recruitment of T cells. *Circulation*.

[36] Kralev S., Schneider K., Lang S., Süselbeck T., Borggrefe M. (2011). Incidence and severity of coronary artery disease in patients with atrial fibrillation undergoing first-time coronary angiography. *PLoS ONE*.

[37] Jessup M., Brozena S. (2003). Heart failure. *The New England Journal of Medicine*.

[38] Pearson T. A., Manolio T. A. (2008). How to interpret a genome-wide association study. *Journal of the American Medical Association*.

